# Inconsistent values and algorithmic fairness: a review of organ allocation priority systems in the United States

**DOI:** 10.1186/s12910-024-01116-x

**Published:** 2024-10-17

**Authors:** Reid Dale, Maggie Cheng, Katharine Casselman Pines, Maria Elizabeth Currie

**Affiliations:** Department of Cardiothoracic Surgery, Stanford University School of Medicine, Center for Academic Medicine, 453 Quarry Road, Room 267, MC 5661, Stanford, CA 94304 USA

**Keywords:** Transplantation, Organ allocation, Model for End-Stage Liver Disease (MELD), Kidney Donor Risk Index (KDRI), Kidney Donor Profile Index (KDPI), Estimated Post Transplant Survival (EPTS), The Lung Allocation Score (LAS), Heart Tier System

## Abstract

**Background:**

The Organ Procurement and Transplant Network (OPTN) Final Rule guides national organ transplantation policies, mandating equitable organ allocation and organ-specific priority stratification systems. Current allocation scores rely on mortality predictions.

**Methods:**

We examined the alignment between the ethical priorities across organ prioritization systems and the statistical design of the risk models in question. We searched PubMed for literature on organ allocation history, policy, and ethics in the United States.

**Results:**

We identified 127 relevant articles, covering kidney (19), liver (60), lung (24), and heart transplants (23), and transplant accessibility (1). Current risk scores emphasize model performance and overlook ethical concerns in variable selection. The inclusion of race, sex, and geographical limits as categorical variables lacks biological basis; therefore, blurring the line between evidence-based models and discrimination. Comprehensive ethical and equity evaluation of risk scores is lacking, with only limited discussion of the algorithmic fairness of the Model for End-Stage Liver Disease (MELD) and the Kidney Donor Risk Index (KDRI) in some literature. We uncovered the inconsistent ethical standards underlying organ allocation scores in the United States. Specifically, we highlighted the exception points in MELD, the inclusion of race in KDRI, the geographical limit in the Lung Allocation Score, and the inadequacy of risk stratification in the Heart Tier system, creating obstacles for medically underserved populations.

**Conclusions:**

We encourage efforts to address statistical and ethical concerns in organ allocation models and urge standardization and transparency in policy development to ensure fairness, equitability, and evidence-based risk predictions.

## Background

The Organ Procurement and Transplantation Network (OPTN), administered by the United Network for Organ Sharing (UNOS) since 1986, guides organ allocation and distribution within the United States [1]. The OPTN Final Rule (herein “Final Rule”), effective on March 16th, 2000, establishes the structure for equitable organ allocation policies [2]. Previously, organs were distributed within limited geographic areas. The Final Rule dictates that organs must be offered for transplant on a national scale should an urgent match not be available within a limited geographic region [3]. While all solid organ transplants are united under the Final Rule, each organ subcommittee determines the classification of transplant recipient urgency and subsequent allocation scores for its respective organs. Organ allocation scores are prediction models based on observed data and are used to numerically prioritize patients based on patient characteristics. Currently, the allocation scores for solid organ transplants include Model for End-Stage Liver Disease (MELD) for the liver; the Kidney Donor Risk Index (KDRI), Kidney Donor Profile Index (KDPI), and Estimated Post Transplant Survival (EPTS) used in conjunction for the kidney; the Lung Allocation Score (LAS) for the lung; and the Heart Tier System for the heart.

One drawback to this organ-specific framework is the complexity in the ethical priorities of the allocation systems for each organ. For example, the MELD score was developed as a model of medical urgency which prioritizes the most acute patients. In contrast, the EPTS score prioritizes postoperative life expectancy and is used in conjunction with KDPI to minimize the likelihood of retransplantation. Complicating matters further, adjustments have been made to these scores since their initial deployment to correct for equity issues such as sex and biological compatibility [4–8].

The lack of ethical uniformity across UNOS allocation policies makes it difficult to assess the adequacy of a given score to achieve its stated objective. Moreover, analysis of transplant allocation scores is obscured by the fact that each score may be trying to achieve multiple objectives at once, all of which may be in conflict.

When the ethical justification for the use of a score is given, reports regarding the predictive performance of scores are mixed. It is insufficient to conclude that because a score is predictive of some outcome, that particular score is justified in its implementation for organ allocation. Clear and consistent guidelines for the development and validation of scores across solid organs must be developed and adopted to provide transparency and facilitate more meaningful dialogue around organ allocation policies. Here, we present a systematic literature review of the current state of organ transplant allocation scores in liver, kidney, lung, and heart, focusing on score designs, their statistical performance, and ethical considerations.

## Methods

### Search strategy

This systematic review was conducted according to the Preferred Reporting Items for Systematic Reviews and Meta-Analyses (PRISMA) guidelines. We searched PubMed for published articles on organ allocation risk scores and algorithmic fairness to explore the allocation of liver, kidney, lung, and heart. Keywords and search terms included “organ allocation scores”, “continuous distribution”, “predict* AND organ allocation”, “fairness AND organ allocation”, “allocation policy equity”, “liver allocation AND MELD”, “Lung Allocation Score”, “Heart Tier System”, “donor heart allocation”, “KDRI and race”, “kidney transplant allocation”, and “organ allocation ethics.” The literature search concluded on September 4th, 2023.

### Inclusion and exclusion criteria

We included published literature discussing adult organ allocation in the United States. All article types (observational studies, reviews, editorials, opinion articles, policy briefs, etc.) were considered and screened. Exclusion criteria included records on (1) multi-organ transplantation, (2) re-transplantation, (3) pediatric organ transplantation, (4) organ allocation outside of the United States, (5) non-English publications, (6) end-stage organ failure discussion without reference to organ allocation, (7) review of surgical procedures of transplantation, (8) records that are irrelevant to organ transplantation, (9) records without access to abstract and/or full text, and (10) other articles outside of the scope of this review. Examples of out-of-scope records include topics like (1) delisting candidates from the waitlist, (2) discussions of fairness without reference to organ allocation risk scores, (3) previously addressed issues in the allocation system, and (4) previously proposed allocation algorithms that were not adopted.

## Results

### Summary of search results and record screening process

**Fig. 1:**
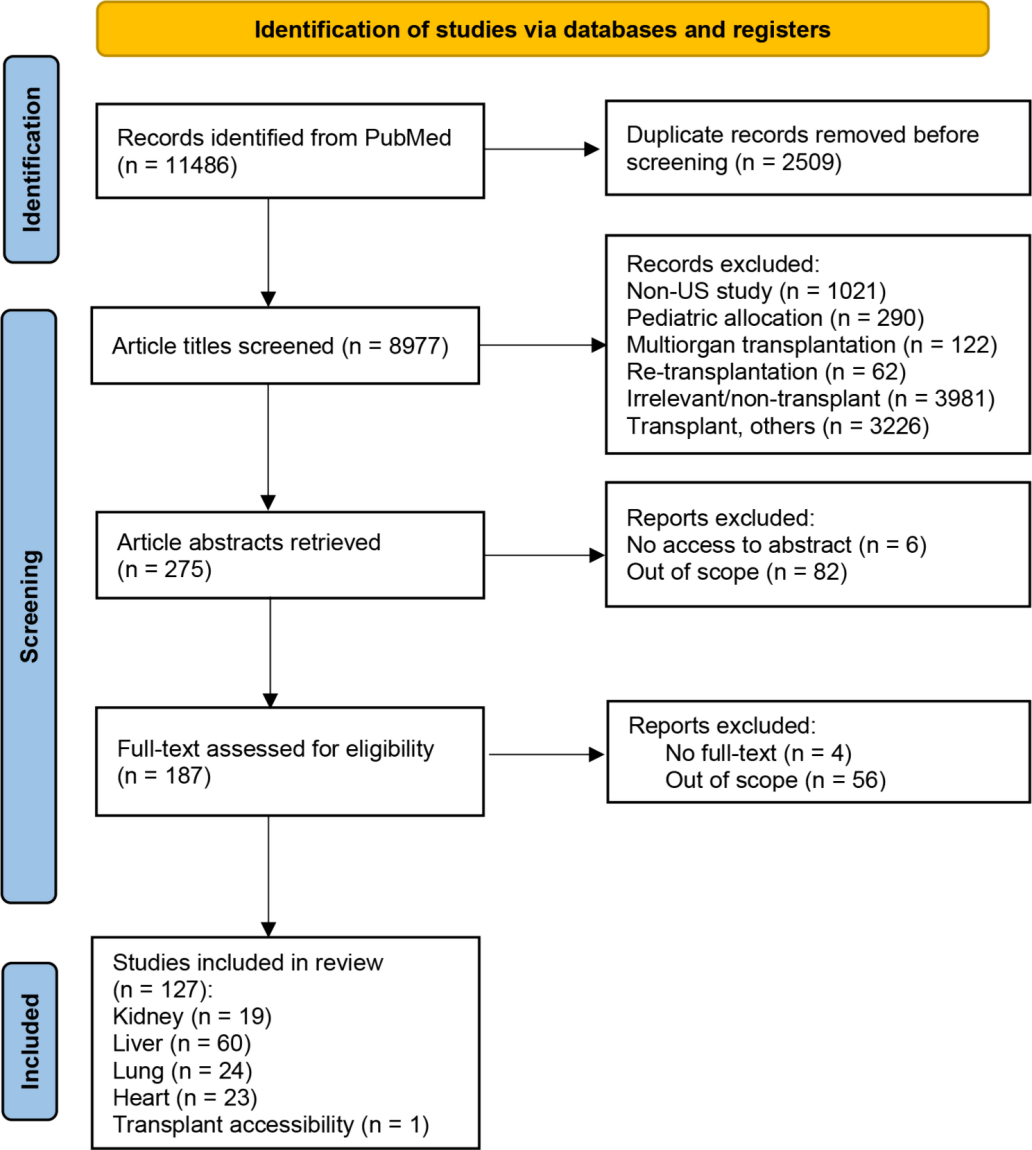
PRISMA flow diagram

A total of 11,486 records were retrieved from PubMed. These included all search results generated from the aforementioned search terms. Records were first screened for duplication (*n* = 2509) followed by a title screen for relevance. Non-US reports (*n* = 1021) and records on pediatric transplants (*n* = 290), multiorgan transplants (*n* = 122), re-transplantations (*n* = 62), irrelevant/non-transplant (*n* = 3981), and transplants (others) (*n* = 3226) were excluded. Transplant (others) records included articles that fit exclusion criteria 6, 7, and 10. Records that fit more than one exclusion criteria were only counted into one of the categories. Next, article abstracts (*n* = 275) were retrieved. Six records were excluded due to lack of access to the abstract, and 82 records were excluded for being outside of the scope of this review. Afterward, full-text screening of the remaining records (*n* = 187) was performed with 4 excluded for lack of full-text and 56 excluded for being out of scope. The PRISMA diagram is shown in Fig. 1.

Ultimately, 127 records were included in the current review. These included 19 records on kidney allocation, 60 on liver allocation, 24 on lung allocation, 23 on heart allocation, and 1 on transplantation accessibility. Records on continuous distribution and allocation fairness were categorized according to the specific organ category they discussed.

### Overview of organ allocation schema based on predictive models

#### Liver transplant allocation

The liver allocation policy has changed significantly from the time-accrual system to the Child-Turcotte-Pugh (CTP) score to the MELD system (see Table 1), with each iteration increasing objectivity in recipient risk assessment [9–35].

**Table 1:** History and development of liver allocation

Name	Contributing Author(s)	Year of Publication	Year of Adoption by UNOS	Objective	Included Criteria	Changes to Criteria	Range of Scores	Calculation
Before CTP	N/A	N/A	N/A	Predict death without transplant Identify highest severity	Severity of liver disease, time accrued on the waitlist	N/A	N/A	Severity of disease was reflected by the level of care received: hospitalized (ICU), hospitalized (general), outpatient care
Child–Pugh–Turcotte (CTP) score	Pugh et al.	1973	1996	Prioritize sicker patients for liver transplantation	Serum albumin, serum bilirubin, prothrombin time, ascites, encephalopathy	+ Serum Albumin + Serum Bilirubin + INR + Ascites + Encephalopathy - Time accrued on waitlist - Treatment and hospitalization status	5 to 15	Each variable was scored from 1 to 3 and was given equal weight. Individual scores were added together for a final CTP score
Transjugular intrahepatic portosystemic shunt (TIPS) Model	Malinchoc M, Kamath PS, Gordon FD, Peine CJ, Rank J, ter Borg PC. A model to predict poor survival in patients undergoing transjugular intrahepatic portosystemic shunts. Hepatology. 2000;31:864–871.	2000	N/A	Predict three-month mortality in cirrhotic patients undergoing TIPS	Serum creatinine, serum bilirubin, INR, etiology of cirrhosis	+ Serum Creatinine + etiology of cirrhosis - Ascites - Encephalopathy		TIPS risk score = 0.957 x ln(creatinine mg/dL) + 0.378 x ln(bilirubin mg/dL) + 1.120 x ln(INR) + 0.643 x (cause of cirrhosis).
MELD	Kamath et al.	2001	2002	Predict survival	Serum bilirubin, creatinine, INR	+ Creatinine - Albumin - Ascites - Encephalopathy	6 to 40	MELD = 9.57 x ln (creatinine) + 3.78 x ln (total bilirubin) + 11.2 x ln (INR) + 6.43
MELD-Na	Kim et al.	2008	2014	Predict survival	Serum bilirubin, creatinine, INR, sodium	+ Sodium	6 to 40	MELD-Na = MELD + [1.32 × (137 - Na) - [0.033 x MELD x (137 - Na)]
MELD 3.0	Kim et al.	2021	2023	Predict survival	Serum bilirubin, albumin, creatinine, INR, sodium, sex	+ Albumin + Sex	6 to 40	MELD 3.0 = 1.33 (if female) + [4.56 x In (bilirubin)] + [0.82 × (137 - Na)] - [0.24 × (137 - Na) x In (bilirubin)] + [9.09 x In (INR)] + [11.14 x In (creatinine)] + [1.85 × (3.5 - albumin)] - [1.83 × (3.5 - albumin) x ln (creatinine)] + 6

All variants of the MELD score characterize recipients’ disease severity and medical urgency; [2] the objective is to reduce waitlist mortality rather than prioritizing individuals with the best predicted post-transplant survival [36–38]. Under MELD, patients with higher MELD scores may receive transplants before those with lower scores despite potentially worse post-transplant survival [39, 40]. The current liver allocation score is the MELD 3.0, implemented in 2023 [41–43].

#### Ethical challenges facing MELD

Some adjustments to the MELD score seem to contradict the initial intention of developing MELD as a purely objective reflection of disease severity. For example, MELD exception points were granted to patients with hepatocellular carcinoma to increase their likelihood of transplantation, as the severity of their disease was believed to be inadequately reflected by variables included in MELD [25, 44]. Sex was added as a corrective factor in MELD 3.0 to award an additional 1.33 points for the female sex. Previously, female patients were removed from the waitlist for fragility and disadvantages in score calibration due to lower muscle mass resulting in lower creatinine-related MELD points. (41–42, 44, 7, 8, 45, 6, 5, 46, 4) This was also justified based on the fact that females had increased mortality rates, psychological distress, and poorer health-related quality of life post-liver transplantation [5–8, 45, 47]. The original MELD score was identified as a factor to unintentionally lower liver transplantation rates in women, and this disparity was worsened by MELD-Na. (45–6, 4, 47, 48) Initial studies on the effects of MELD 3.0 showed that the addition of sex in the model not only addressed sex disparities for liver transplantation but also had population-level benefits by reducing waitlist deaths by at least 20 per year [41].

#### Statistical challenges facing MELD

MELD 3.0 improved the predictability and discriminatory abilities of the model compared to the previous MELD-Na and MELD scores. Utilizing data from adult liver transplant candidates without prior transplant history from January 15, 2016, to December 31, 2018, Kim et al. revealed a statistically significant c-statistic difference of 0.869 for MELD 3.0 versus 0.862 for MELD-Na for 90-day mortality prediction [41].

Although MELD was effective in predicting 3-month mortality post-transplant, the model suffers from its static setup and lack of flexibility [49–51]. The discriminatory and predictive powers of MELD and MELD-Na decreased with time due to changing etiologies of end-stage liver diseases [33, 49–51]. A more dynamic model that can adjust with time could be effective in addressing this limitation.

The statistical consequences of MELD exception points raise concerns. Northup et al. showed that patients with hepatocellular carcinoma who received exception points in the liver allocation process had better post-transplant outcomes than those without exception points [52, 53]. This warrants further investigation in the use of 1.33 additional points in MELD 3.0 and calls for a re-evaluation of exception points in models to avoid over-correction.

#### Alternatives to MELD – the optimized prediction of mortality score

As a linear regression model that assigns different weights to different variables in the equation, MELD may not accurately reflect the mortality of all populations [54]. Bertsimas et al. developed a new model in 2018, the Optimized Prediction of Mortality (OPOM), using machine learning and variables in MELD [54]. The OPOM was tested using the Liver Simulated Allocation Model from the Scientific Registry of Transplant Recipients (SRTR). Bertsimas et al.’s analysis found that OPOM reduced mortality and increased survival in all populations compared to MELD, therefore more objectively, accurately, and equitably prioritizing patients for liver transplantations. (54–55) However, the objectivity of machine learning model algorithms may perpetuate disparities if they exist in the training dataset. Therefore, caution should be used when implementing algorithms to minimize such bias.

MELD 3.0 was developed three years after OPOM and was approved for use instead due to its verifiability, generalizability, and easy-to-understand formulation [56]. The OPOM lacks validation from researchers other than those who developed it, and the complexity of the algorithm limits its transparency.

#### Kidney transplant allocation

The policy for decreased kidney allocation has shifted away from merely considering waitlist time and human leukocyte antigens matching. The current Kidney Allocation System, implemented in 2014, utilizes KDRI, KDPI, and EPTS to consider both donor and recipient risk factors in the allocation process (Table 2) [57–63]. 

**Table 2:** Risk scores of current deceased kidney allocation

Name	Contributing Author(s)	Year of Publication	Year of Adoption by UNOS	Included Criteria	Scoring method	Calculation
KDRI	Rao et al.	2009	2014	Age, height, weight, race/ethnicity, history of hypertension, history of diabetes, cause of death, serum creatinine, HCV status, DCD status	Range from 0-100th percentile	KDRI_RAO_ = e ^sum of KDRI score components^
KDPI	Unknown	Unknown	2014	(same as above)	Range from 0-100th percentile	Map KDRI scores on a percentile scale to compare the relative risk of a donor organ to other donor organs.
EPTS	Scientific Registry of Transplant Recipients (SRTR) contractor	Unknown	2014	Age, prior history of solid organ transplant, diabetes status, time on dialysis	Range from 0-100th percentile	Raw EPTS score = 0.047 x max(Age − 25, 0) − 0.015 x Diabetes x max(Age − 25, 0) + 0.398 x Prior Solid Organ Transplant − 0.237 x Diabetes x Prior Organ Transplant + 0.315 x log(Years on Dialysis + 1) − 0.099 x Diabetes x log(Years on Dialysis + 1) + 0.130 x (Years on Dialysis = 0) − 0.348 x Diabetes x (Years on Dialysis = 0) + 1.262 x Diabetes

The KDRI assesses the risk of donor graft failure [50]. The KDPI then maps the KDRI score on a percentile scale, reflecting the relative quality of the donor organ, with a lower KDPI percentile corresponding to lower chances of graft failure. The EPTS considers recipient characteristics, with a lower score reflecting longer predicted post-transplant survival [64]. EPTS is used when the donor has a KDPI of ≤ 20% to optimize matching of lower-risk donor organs to recipients with longer projected post-transplant survival, therefore maximizing the realized benefit of high-quality donor organs [59–70].

### Ethical challenges facing KDRI and EPTS

#### The use of race as a variable

The initial KDRI assigned a hazard ratio of 1.196 to black donors [58, 71]. This implied that kidneys from deceased black donors have a higher risk of graft failure even when all other factors are the same, which leads to a higher organ discard rate for these donors [72]. The inclusion of race as a predictive variable is controversial and poorly justified, based only on epidemiological data showing a consistent graft failure trend among donors of the same racial group with no identified physiological cause of adverse post-transplant survival [73]. Attempts have been made to eliminate or replace the race variable with a more justifiable criteria. Julian et al. replaced race in the model by suggesting the incorporation of the apolipoprotein L1 (APOL1) genotype instead, arguing that the APOL1 genotype might be a better predictor of graft failure than race and the effects of race on the model’s predictivity were no longer significant when the APOL1 genotype was included as a variable [71]. However, subsequent results are mixed: although Julian et al.’s results were confirmed by some later analyses [72], Gill et al. reported that the relationship between APOL1 genotype and post-transplant survival is not confirmed as a uniform trend [73]. APOL1 has yet to be incorporated into the KDRI calculation [74].

#### Longevity matching

Longevity matching with KDPI and EPTS aims to minimize re-transplantation, but this logic does not fully consider the recipients’ quality of life after transplantation and implies that the length of survival is of greater importance [63]. Although freedom from dialysis can potentially lead to improved quality of life, adjustment to post-transplant life and the implications of receiving life-long immunosuppressive therapy should also be considered. Utilizing this longevity matching mechanism for only the top 20% of donor organs and recipients risks ignoring patients with less optimal predicted survival [66]. The cut-off of 20% warrants more careful evaluation, and further research should explore the determination of this cut-off to ensure model sensitivity as well as potentially considering a similar mechanism for those near the bottom of the waitlist [68].

#### Statistical challenges facing KDRI and EPTS

KDRI is not the uniquely best predictor of waitlist mortality or post-transplant survival for kidney transplantation. Replacing race with the APOL1 genotype or simply removing race in KDRI was effective with similar model predictivity. This questions the equitability and statistical significance of including race in the KDRI model [71, 72, 75].

By studying renal-risk variants of the APOL1 gene in black deceased donors (*n* = 1149) from the SRTR database, Julian et al. found that the APOL1-adjusted version of KDRI recategorized around 85% of the kidneys from black donors to a less hazardous category, allowing more kidneys for standard allocation [71]. The two models also showed no statistically significant differences in predictive and discriminatory powers when it comes to graft survival prediction [71]. A similar study by Miller et al. with a larger sample size (*n* = 72,926) of SRTR data from 2015 to 2021 found the same results [73].

Doshi et al. further assessed the predictability and discriminatory power of the race-free model by simply zeroing out the race adjustment factor in KDRI [75]. Analysis of the SRTR data with 66,987 decreased-donor kidney transplants performed between January 1, 2010, and December 31, 2016, in the United States showed that the risk discrimination and c-statistic of the model were only minimally affected by removing the race variable. When it comes to graft failure predictions, the race-based KDRI had a c-statistic of 0.640, and that for the race-free KDRI was 0.639, only a 0.16% decrease.^80^

Therefore, the race-free KDRI not only increased the objectivity of the model by using physiological variables but also increased organ utility and availability and reduced discard with no compromise on the predictive ability of the model.

#### Lung transplant allocation

As with other organs, lung allocation underwent multiple policy and score changes prior to the implementation of the Final Rule [76]. The current Lung Allocation Score (LAS) was implemented in the United States in 2005 [77]. The LAS was designed to improve access and equity of lung distribution by replacing subjective components with laboratory measurements [77–82].

The LAS was accepted and widely used due to its increased equitability and objectivity compared to the previous time-accrual system [83]. It also increased transplant volume and decreased wait time and waitlist mortality compared to the previous model [84–91]. Takahashi et al. reported a reduction in the number of patients waitlisted for lung transplants by 54%, the median waitlist time shortened from almost three years to less than 141 days, and waitlist mortality decreased by 31% from pre-LAS in 2004 to post-LAS in 2007 [88]. The LAS also excluded variables like waitlisted time, which could be easily affected by either the patient or the provider.

### Ethical challenges facing LAS

#### Post-transplant survival

LAS balances waitlist mortality and post-transplant survival by taking the weighted average of two sub-scores: the Waitlist Area Under Curve (WLAUC) and the Post-Transplant Area Under Curve (PTAUC) [79, 82, 92] (see Table 3). However, these two objectives can be hard to achieve simultaneously. Patients with a high LAS (the “sickest”) may be prioritized for lung transplantation, but that does not necessarily guarantee longer survival post-transplant compared to remaining on the waitlist. At the same time, emphasizing a reduction in waitlist deaths may shift organ supplies to more critical patients with potentially shorter post-transplant survival, leading to lower realized benefits from the donor organ and a higher discard rate, affecting everyone on the waitlist [80, 93, 94].

**Table 3:** History and development of lung allocation

Name	Contributing Author(s)	Year of Publication	Year of Adoption by UNOS	Objective	Included Criteria	Changes to Criteria	Range of Scores	Calculation
N/A	N/A	Unknown	Unknown	Balance postoperative survival and risk of death without transplant	Time accrued on the waitlist	N/A	N/A	N/A
N/A	UNOS	N/A	1995	Balance postoperative survival and risk of death without transplant	Time accrued on the waitlist, idiopathic pulmonary fibrosis (IPF) status	+ IPF status	90 days were added to patients on the waitlist who had IPF	N/A
Lung Allocation Score	UNOS Thoracic Organ Transplantation Committee Lung Allocation Subcommittee	Unknown	2005	Balance postoperative survival and risk of death without transplant	Age, assisted ventilation requirement, serum, bilirubin and creatinine, body mass index, cardiac index before exercise, central venous pressure, mechanical ventilation status, diabetes status, diagnosis, forced vital capacity, functional status, oxygen requirement at rest, pulmonary artery pressure, 6-min walk test distance	*All variables are added compared to the previous model. Variables in the previous model no longer exist in this version	The waitlist and post-transplant area under the curve are mapped. Transplant benefit is calculated as the difference between post-transplant and waitlist survival. The normalized LAS score ranges from 0 to 100.	Raw LAS = posttransplant survival − 2 x (waitlist urgency measure). Normalized LAS = 100 x [raw LAS x (2 × 365)]/(3 × 365).
Composite Allocation Score	OPTN ad hoc Committee on Geography	2019	2023	Balance postoperative survival and risk of death without transplant	Waitlist survival (5-year WLAUC), post-transplant survival (5-year PTAUC), biological disadvantages (blood type, CPRA, height), patient access (prior living donation, pediatric status), placement efficiency (travel cost and proximity)	+ length of WTAUC and PTAUC assessments (1 year vs. 5 years) + prior living donation status + pediatric status	From 0 to 100	CAS = 0.25 x waitlist survival + 0.25 x post-transplant survival + 0.15 x biological disadvantages + 0.25 x patient access + 0.1 x placement efficiency

#### Geographic barriers

In addition, the LAS prioritizes patients within 250 nautical miles to the donor [95]. Consequently, Russo et al. revealed that nine out of ten donor lungs are actually allocated to lower-priority candidates, potentially due to this geographical preference [96]. Residential areas and the ability to move for transplantation vary by recipient and are largely affected by their socioeconomic status and social network. Although setting a geographical limit allows shorter cold ischemic time for donor organs, less travel and carbon footprint for the procurement team, and lower costs and fewer hospital stays given the recipients are of lower LAS at transplantation on average, it can have unintended consequences [89, 94]. Setting geographical boundaries may indirectly and disproportionately harm patients who already face barriers to health services (e.g., low socioeconomic status, language barriers, transportation concerns), worsening existing health inequities [80, 93, 94].

#### Statistical challenges facing LAS

Multiple studies have questioned the predictive accuracy of the LAS, demonstrating that the score was only mildly predictive of post-transplant outcomes and had poor discriminatory power [77, 84, 96]. Parker et al. assessed the accuracy of WLAUC and PTAUC by comparing predicted and observed values for each score in a retrospective study with data from SRTR (11,539 lung transplant candidates and 9,377 recipients age 12 or above from February 19, 2015, to February 19, 2019) [84]. By comparing predicted outcomes to observed outcomes, they revealed clinically significant errors in both WLAUC and PTAUC calculations based on the LAS. WLAUC reflects medical urgency but significantly underestimated waitlist survival, with increasing inaccuracy as the LAS score increased [84]. Looking at overall data, the WLAUC correctly ranked 72% of patients and had a c-statistic of 0.85 for the prediction of waitlist survival without a transplant [84].

A similar trend is observed in utility prediction by PTAUC. Post-transplant survival of the low-risk group was underestimated by 10 days while that of the high-risk group was underestimated by 70 days [84]. PTAUC performed even worse than WLAUC with a correct ranking of 57% of patients and a c-statistic of 0.58 for post-transplant survival [84]. The predicted LAS poorly explained variations in the observed LAS with an r [2] value of only 0.56 [84]. The poor ability of the LAS to predict post-transplant survival may be attributed to the lack of donor factors in the prediction formula [77, 84]. The increasingly worse predictive and discriminatory ability of the model toward high scores is especially problematic, as it largely underestimates the potential benefits the sickest patients may receive from lung transplantation and excludes them from the top of the transplant list [97]. Candidates with low LAS scores may have better survival rates remaining on the waiting list, but received a transplant due to geographic advantages, resulting in negative or no survival benefits. Those on the high end of the risk scale may have low predicted waitlist survival. However, their post-transplant outcome may also be poor due to their high LAS [96].

The LAS also only predicts survival one year after transplant. Maxwell et al. offered a possible explanation that one-year survival is commonly used to evaluate transplantation programs [98]. Maxwell et al. also identified that one-year post-transplant survival was worse after LAS implementation compared to before implementation [98]. Whether the predictive power and discriminative ability of the LAS still hold beyond one year is unclear and is an area of further research.

#### Heart transplant allocation

Three heart allocation systems have been used by UNOS: The first, implemented in 1988, was a two-tiered system that assessed individual risks and assigned transplantation priority; the second, implemented in 1998, added a third tier and provided more specific guidelines for waitlist placement; the third and current model, implemented in 2018, grew to a six-tier system that stratifies individual risks and increases transplantation access for the sickest (see Table 4 for all version tier criteria) [99–103].

**Table 4:** History and development of Heart allocation (Heart Tier System)

Name	Contributing Author(s)	Year of Publication	Year of Adoption by UNOS	Objective	Categories	Changes to Categories
Heart Tier System (2 tiers)	N/A	N/A	1988	Stratify recipient risks based on clinical treatment	Used for both adults and children. Status 1: 1) Individuals with an implanted mechanical circulatory support (MCS) device including an intra-aortic balloon pump (IABP), total artificial heart (TAH), ventricular assist device (VAD) 2) Individuals who are continuously intubated 3) Individuals admitted to the intensive care unit for inotropic support. 4) Individuals younger than 6 months. Status 2: All other patients eligible for transplant.	N/A
Heart Tier System (3 tiers)	N/A	N/A	2005	Stratify recipient risks based on clinical treatment	Status 1 A: 1) Individuals with an implanted mechanical circulatory support (MCS) device including an intra-aortic balloon pump (IABP), total artificial heart (TAH), and extracorporeal membrane oxygenation (ECMO) 2) Individuals on continuous inotropic support and hemodynamic monitoring 3) Individuals with dischargeable VADs (> 30 days) or VAD complications 4) Individuals on continuous mechanical ventilation Status 1B: 1) All other individuals who are stable on LVAD/RVAD 2) Individuals on continuous IV inotropic support without hemodynamic monitoring Status 2: All other patients eligible for transplant.	Increased in priority: ↑ N/A Decreased in priority: ↓ All other individuals who are stable on LVAD/RVAD ↓ Individuals on continuous IV inotropic support without hemodynamic monitoring
Heart Tier System (6 tiers)		2018	2018	Stratify recipient risks based on clinical treatment	Status 1: 1) ECMO for up to 7 days 2) Implanted, non-dischargeable BiVAD 3) MCS device with life threatening ventricular arrythmias Status 2: 1) Dischargeable BiVAD/RVAD 2) Non-dischargeable LVAD, IABP, or percutaneous MCS for up to 14 days 3) MCS device failure 4) Total artificial heart Status 3: 1) Dischargeable LVAD for up to 30 days 2) Individuals on continuous inotropic support and hemodynamic monitoring 3) ECMO after 7 days 4) Non-dischargeable LVAD, IABP, or percutaneous MCS devices after 14 days 5) MCS devices with complications Status 4: 1) Stable on LVAD 2) Individuals on continuous IV inotropic support without hemodynamic monitoring 3) Congenital Heart Disease 4) Re-transplant 5) Restrictive cardiomyopathy 6) Amyloidosis 7) Hypertrophic myopathy 8) Ischemic heart disease with recurrent angina Status 5: 1) Multiorgan transplant Status 6: All other patients eligible for transplant.	Increased in priority: ↑ N/A Decreased in priority: ↓ Stable on LVAD ↓ Individuals on continuous IV inotropic support without hemodynamic monitoring

The current six-tier system improved from the previous models in three ways. First, it expanded the geographical barriers that existed in previous models, allowing donor and recipient matches within 500 nautical miles [104]. This partially addressed the issue of lower-priority patients within the Donor Service Area (DSA) receiving transplantation before those of a higher priority who reside outside of the DSA, therefore improving transplantation access [99, 100]. Second, the volume of heart transplantation performed after the policy changes in 2018 increased and the overall waitlist mortality decreased [100, 105]. Third, a larger proportion of patients at the highest listing status received transplants, achieving the goal of the policy change to prioritize the sickest patients [100].

However, these benefits were not without consequences. In the six-tier system, the three-month post-transplant survival probability was estimated to be 87.6% and the six-month survival was approximately 77.9%, compared to 94.5% and 93.4%, respectively, in the three-tier system; both differences were statistically significant [99, 105]. This may be attributed to longer ischemic time, increased donor and recipient ages, greater usage of short-term mechanical circulatory support devices, and the high hazard rate for death or retransplantation among those in the highest risk category [105]. This suggests a potential increase in graft loss and low realized benefits from the donor organ. Moreover, this calls into question whether the reduction in waitlist mortality outweighs the worsened patient outcome after transplantation and whether this policy change was indeed beneficial [106–111].

### Ethical challenges facing the heart tier system

The modifications in tier ranking led to changes in clinical behaviors. The utilization of left ventricular assist devices has decreased significantly since the adoption of the six-tier system, as patients on these devices without complications are now ranked at a lower priority status than before [101, 104]. The usage of short-term mechanical circulatory support (namely intra-aortic balloon pumps and extracorporeal membrane oxygenation) has increased significantly since the policy change in 2018 [112–117]. This can partially be attributed to the higher status ranking associated with such device usage [101, 107].

These outcomes reveal the highly subjective nature of listing and prioritization of heart allocation. These observed clinical behavioral changes following policy modifications suggest that the system is still vulnerable to manipulation. While the six-tier system requires some physiological criteria to meet tier indications, status assignment is still largely dependent on treatment and device usage rather than intrinsic risk factors or absolute disease severity [99]. The decisions of the transplant center may also be considered in this situation. Transplant centers may be disincentivized to transplant high-risk cases, due to possible penalization if the procedure does not have a favorable outcome. Requests for exceptions remain high after the policy modification, although exception requests are now processed by a different region from which they were originally submitted to minimize bias [100].

Although the current six-tier system aims to improve transplantation access to all patients regardless of socioeconomic background and residency, the system design does not reflect this purpose. The partially subjective nature of the ranking system as well as the current OPTN policy allowing patients to be listed at different centers with different wait times incentivizes candidates to relocate to obtain a more favorable ranking, despite increases in geographical sharing [99, 118]. This not only violates the equitable access principle in the Final Rule but also disproportionately disadvantages those of lower socioeconomic status who cannot afford expenses related to multi-listing.

### Statistical challenges facing heart tier system

The heart tier ranking system is based on risk stratification. The change from a three-tier to a six-tier system was intended to further break down heterogeneity within status and improve risk stratification [119, 120]. However, it is unclear whether the six-tier system is sufficient to achieve this purpose. If heterogeneous risk groups exist in the three-tier system, then it is reasonable to assume that these groups continue to exist in the six-tier system, although perhaps less prominently. With the tier system, many continuous variables are treated as categorical ones, blurring the risk difference between individuals [121].

The discriminatory power of current risk scores for posttransplant outcomes is limited. The c-statistic of the current heart tier system ranges from 0.544 to 0.646 with a median of 0.594. Furthermore, many overlapping categorizations exist among individuals with and without post-transplant mortality [121].

### The development of the continuous distribution system

Current organ allocation risk scores have different criteria and priorities in their development, although many of them share similar limitations. For example, categorical variables such as sex and race are considered in the same way as continuous variables in risk score calculations; evidence on geographical sharing is mixed [122], and geographic parameters limit access to organ transplantation despite policies to encourage broader sharing; multiple listing is allowed [118, 123–126], benefiting those with the resources to relocate; risk score designs are largely static, making model adjustments challenging to update with changing clinical needs; and all risk scores except those for the kidney ignore donor-specific criteria [127], overlooking half of the allocation equation.

Continuous distribution models are an emerging effort to address limitations in current organ allocation risk scores. The concept of continuous distribution was initially proposed by the OPTN ad hoc Committee on Geography and was approved by the OPTN Board of Directors in December 2018 [128]. The experimentation with the continuous distribution system started with lung allocation. To improve the LAS, a composite allocation score (CAS) was developed and was approved for clinical use on March 9, 2023. Six components are included in the CAS: waitlist survival (WLAUC, similar to the LAS calculation), post-transplant survival (based on 5-year PTAUC), candidate biology (blood type, calculated panel reactive antibody, and height), pediatric status (binary, with additional points for pediatric patients), prior organ donation (binary, with additional points for living donors), and placement efficiency (proximity and travel cost) [95]. Compared to the LAS, which has an arbitrary cut-off point that defines geographical limitations, the CAS considers distance from the donor alongside other characteristics. By eliminating rigid geographic barriers, the CAS is predicted to decrease variability in transplant volume and waitlist death for all centers and regions. This indirectly lessens the effects of center-specific characteristics that may lead to differential selection and transplantation as previously mentioned [92]. In addition to balancing the predictability and equitability of the model, stakeholders’ feedback was also incorporated into the development process [95, 129].

In addition to removing geographic barriers to transplantation access, the continuous distribution system also allows more uniformity in allocation across the different organs. Parameters may be continuously adjusted to reflect ethical and public priorities, and improvement in one organ allocation area may inform and facilitate the prompt adjustment of others [130].

## Discussion

### Relationship between prediction and allocation

The cases of MELD and KDRI show the tension in risk score development and organ allocation processes: should risk scores prioritize the most acute cases, like MELD, and reduce waitlist mortality or should they aim to maximize total benefits, as in KDRI/EPTS, and ensure the maximization of life years gained overall? Regardless, both MELD and KDRI emphasize using standard measurements to predict post-transplant outcomes to some extent.

We question whether better outcomes alone are the most significant determinant of prioritization and allocation. If so, what about social and ethical responsibilities in the allocation process? If better predictability alone justifies the use of risk scores in organ allocation, then how should variables be selected to ensure fairness and equity of allocation while maximizing outcomes?

In current risk scores like MELD or KDRI, the inclusion of many predictive variables is not sufficiently justified. Revisiting the race-based KDRI example, race was included as a variable due to the observed association between racial groups and post-transplant outcomes. However, such observation was later found to be equally well-accounted for by the APOL1 genotype rather than socially constructed race [71]. Therefore, although using race in the risk score calculation may give a similar aggregate predictive performance, allocating based on race discriminates against non-physiological factors. Withholding treatment or deprioritizing organs due to non-causal factors is not justifiable. The use of proximal causal factors rather than distal ones should be considered when designing processes to allocate resources or treatment [131].

### Disentangling prediction from allocation

Current organ allocation risk scores emphasize mortality predictions and are justified to use in practice due to satisfactory model performance in those areas. However, we encourage greater scrutiny for algorithmic designs and subsequent adjustments and question whether those predictive models are equitable and ethically acceptable for use in the allocation process.

Although both MELD 3.0 and APOL1-based KDRI have similar, if not better, predictive and discriminatory power compared to the previous versions of the models, the logic behind the model adjustment differs.

The addition of sex to MELD 3.0 was used to correct for the observed disparities in liver transplantation allocation and post-transplantation risks between sexes. However, in the scope of this review, we did not identify any subgroup analysis of the predictive performance of the model between sexes other than the observed disparity in transplant outcomes. It is not immediately evident whether there was anything to correct in the model from a predictive standpoint or whether the points addition for female sex was effective. Even if there were no differences in the predictive performance of existing models between sexes, that does not mean that sex should not in some way factor into the allocation system on equity grounds. To use MELD (or any other predictive score) as the *sole* determinant of the waitlist entails difficulties in model generation: simultaneously trying to be as predictive as possible among all subgroups while also being tasked with solving exogenous problems such as equity in allocation. This tradeoff can be avoided by explicitly recognizing that a predictive score can merely be input into a broader allocation schema, preserving the integrity of the score’s design while allowing other facets of the organ allocation system to address the problems of allocative justice.

By contrast, the use of race in KDRI is counterproductive and not well-justified in addressing differences in health outcomes among groups. Differences in health outcomes among racial groups should be attributed to socioeconomic status, health policy, and access and barriers to resources, rather than innate genetics. As seen in the case of KDRI, just because the race-based and race-free models have similar predictive power does not mean that both should be used in clinical practice to allocate organs [42]. When considering the admissibility of a model, equity issues should also be taken into account. Fairness metrics, such as statistical parity differences, and true-positive and true-negative rate differences, should be considered to optimize risk prediction models. A highly fair model should yield the same risk score for two individuals with identical medical profiles and should also apply to any age group [132].

Although the predictability of current risk scores like MELD 3.0 and APOL1-based KDRI is confirmed, the fairness of such models still needs to be carefully evaluated. The selection of variables in current risk prediction models do not clearly show causal effects on outcome. Selection algorithms that have insufficient subject knowledge may introduce bias to the model by mistaking causal factors for associational ones. However, the goals and methods of causation compared to association studies are different, with the first considering confounding factors and the second not [133]. A claim under the association approach may link individual risk factors to transplant outcomes due to individuals with similar covariate makeup having similar outcomes.

Most variables included in risk score models are associational [73], as such a relationship is easy to establish. However, just because one variable is associated with transplant survival does not necessarily warrant its inclusion in prediction models. The association between race and transplant outcomes exists, but it is unclear whether this is due to physiological differences or historical mistreatment and continuous disadvantages faced by certain racial groups. Therefore, clarification of aims, methods, and variable selection are necessary to avoid ambiguity and confusion. Kartoun et al. proposed several ways to improve risk scores, including adjusting the algorithms according to observed unfairness, creating models that include more precisely defined variables, and reweighing existing predictors within each subgroup of the population are also proposed solutions to the issue [134]. However, caution should be taken when selecting and defining predictors to ensure that the model is equitable, objective, verifiable, and transparent [42].

### Fixing prioritization scores will not fix all problems

While risk scores can be optimized to minimize bias and discrimination of certain groups, factors beyond the organ matching and allocation process also contribute to inequities in organ transplantation. Delays and disparities in referrals also contribute to inequitable access to transplantation. Although OPTN policies prohibit organ allocation based on race, ethnicity, or socioeconomic status, the transplantation referral and evaluation processes may be biased against these factors [135]. Black patients are significantly less likely than white patients to be considered suitable transplant recipients and referred for transplant evaluation, even if they express interest in receiving a transplant [136]. People with intellectual and developmental disabilities (IDD) are less than half as likely to be referred for transplantation evaluation or accepted for waitlisting than their counterparts without disabilities [137]. Many cite concerns about post-operative care compliance and quality of life despite evidence suggesting that people with IDD do equally as well post-operatively with adequate support [137, 138]. The differential treatment of certain patient groups further creates barriers to care and establishes trust barriers that may interfere with further clinical interventions. Clinical training and practice should be standardized and adjusted to avoid such bias.

Policies surrounding transplant practice may be another barrier to equitable allocation. The regulatory guidelines around outcome control and center accreditation encourage transplant centers to strive for better postoperative outcomes but may impact the number of high-risk patients a center is willing to waitlist [38, 135]. This disproportionately affects minority and disadvantaged populations as they are more likely to be part of the high-risk group due to public insurance status and limited access to transplant-related care [37, 139].

Although the Final Rule has mandated that socioeconomic status not be considered in the allocation of donor organs, the referral and evaluation processes of transplantation do, which indirectly affects patient access to transplantation services based on socioeconomic status. Ambiguity in policies surrounding patients’ financial security and suitability for transplantation leaves decision-making power in the hands of transplant centers. The desire to avoid potentially adverse outcomes may inhibit transplant centers from accepting patients of low socioeconomic status due to concerns regarding their ability to keep up with post-transplant care. These risk-averse behaviors can be center-specific and may introduce bias that results in different decisions for transplant at different centers, therefore creating inequities in transplant access [135].

### Limitations

This review is subject to the limitations of its sources. The scope of this review is limited to discussing currently adopted organ allocation scores in the United States (Table 5). Many of those scores (e.g., MELD and LAS) are also used in other countries. There are opportunities for larger-scale international studies to review the effectiveness of those organ allocation scores globally. In addition to allocation scores, there are also other aspects in the transplantation processes (referral, evaluation, listing) and allocation (geographic sharing, ABO blood type matching, etc.) that are not reviewed, which could be a topic of future research.

**Table 5:** Comparison of risk scores for each solid organ

Organ	Risk score	Scoring method	Priority	Ethical and statistical concerns
Liver	MELD 3.0	6–40, higher score means more medically urgent	Medical urgency	• The 1.33 additional points for the female sex lacks biological justification and contradicts the purpose of developing MELD as a purely objective score • Lack of flexibility. The discriminatory and predictive powers decreased with time • Not the unique best risk score for post-transplant mortality. Models with similar performance (e.g. OPOM) also exist
Kidney	KDRI/KDPI/ETPS	0-100%, assess graft failure risk compared to the reference recipient	Minimize re-transplantation	• Inclusion of race as a predictive variable • Removing race or replacing it with APOL1 had no significant effect on the model’s predictability • Longevity matching with KDPI and EPTS does not fully consider post-transplant quality of life
Lung	Lung Allocation Score	0-100, higher score means more medically urgent	Balance of post-transplant survival and medical urgency	• Conflict of values between WLAUC and PTAUC • Geographical limit results in local patients with lower LAS receiving transplantation earlier than slightly further patients with higher LAS • Clinically significant error in prediction with increased inaccuracy with higher LAS scores
Heart	Heart Tier System (2018)	Tier 1–6, each with its own qualifying criteria. No stratification within tiers	Medical urgency	• Increases in short-term MCS device usage with changes in scoring criteria • Center-dependent ranking and multi-listing are possible • Insufficient stratification tiers and lack of stratification within tiers • Continuous variables are treated as categorical ones • Very limited discriminatory power

## Conclusions

In conclusion, there is a marked lack of ethical uniformity across organ allocation risk scores in the United States as a result of individual development of scores for each organ type. Current organ allocation scores lack explicit goals and transparent reasoning behind their variable selection. The algorithmic designs of many scores do not accurately reflect the ethical principle underlying their development, and some scores may be statistically satisfactory but carry significant ethical flaws that diminish their suitability in clinical practices. Although organ-specific risk assessment criteria and stratification may be necessary given the differential availability of therapy to manage different end-stage organ diseases, the underlying ethical framework of each risk score and allocation system should be assessed periodically to minimize ambiguity and subjective biases. Therefore, proactive efforts should be initiated to assess the alignment between the ethical and statistical designs of allocation risk scores to ensure rigorousness in both aspects. We also encourage ongoing and future model development and optimization to explicitly identify ethical priorities to improve transparency, social justice, and health equity.

## Data Availability

No datasets were generated or analysed during the current study.

## Abbreviations

APOL1: Apolipoprotein L1
CAS: Composite Allocation Score
CTP: Child-Turcotte-Pugh
DSA: Donor Service Area
EPTS: Estimated Post-Transplant Survival
INR: International Normalized Ratio
KDPI: Kidney Donor Profile Index
KDRI: Kidney Donor Risk Index
LAS: Lung Allocation Score
MELD: Model for End-Stage Liver Disease (MELD)
OPOM: Optimized Prediction of Mortality
OPTN: Organ Procurement and Transplant Network
PTAUC: Post-Transplant Area Under Curve
SRTR: Scientific Registry of Transplant Recipients
UNOS: United Network for Organ Sharing
WLAUC: Waitlist Area Under Curve
